# Experience with a "social model" of capacity building: the Peoples-uni

**DOI:** 10.1186/1478-4491-7-43

**Published:** 2009-05-29

**Authors:** Richard F Heller

**Affiliations:** 1 Peoples Open Access Education Initiative – Peoples-uni, Manchester, UK

## Abstract

**Background:**

Taking advantage of societal trends involving the "third sector", a social model of philanthropy and the open-source software and educational resource movements, provides the opportunity for online education for capacity building at low cost. The Peoples Open Access Education Initiative, Peoples-uni, aims to help build public health capacity in this way, and this paper describes its evolution.

**Methods:**

The development of the Peoples-uni has involved the creation of an administrative infrastructure, calls for and identification of volunteers, development of both the information and communications technology infrastructure and course content, and identification of students and course delivery to them. A pilot course module was offered for delivery.

**Results and Discussion:**

Volunteers have been prepared to become involved in the administrative structures, as trustees, members of advisory and quality assurance and educational oversight groups. More than 100 people have offered to be involved as course developers or as facilitators for course delivery, and to date 46 of these, from 13 countries, have been actively involved. Volunteer experts in information and communications technology have extended open-source course-delivery mechanisms. Following an encouraging pilot course module, 117 students from 23 countries have enrolled in the first set of six course modules. Although the business model is not fully developed, this approach allows current module delivery at USD 50 each, to be more affordable to the target audience than traditional university-based education.

**Conclusion:**

A social model of capacity building in public health has been started and has been able to attract volunteers and students from a wide range of countries. The costs are likely to be low enough to allow this method to make a substantial contribution to capacity building in low-income settings.

## Background

There are a number of societal trends transforming the way people help others, including the development of a "third sector" of the economy in which people are prepared to donate their time and money for the benefit of others [1], and a "social" model of philanthropy in which businesses invest in the hope of a social return on their investment [2]. This latter model had its origins in resource-poor settings, where micro loans through the Grameen Bank have transformed the lives of the poorest of people [3] – and this has been replicated elsewhere.

These trends have not, till now, been applied to the important area of formal capacity building in resource-poor settings. Capacity building can be performed either at a government level to improve the competence of the population as a whole, by the individual who wishes to benefit and work out ways to self-learn, or by institutions that want to improve the capacity of their own employees or of those who will pay them to provide an educational programme of some sort. A variant of the self-learn model can be found as part of the third sector, as in the University of the Third Age, where retired people come together to teach each other on a voluntary basis.

With the exception of this last example, there is usually a high fee involved. In many countries, universities are becoming reliant on fees from overseas students: in Australia, fees from overseas students represent the third largest "export" earner for the country as a whole [4].

Educational programmes aimed at resource-poor settings funded by donors often leave the side benefit of funding for the country providing the education, which may well be used to provide capacity development there as well. These vested interests have come to dominate international capacity building.

This has led the business model to dominate in the education-for-capacity-building sector. Many well-meaning teachers are subverted to meet the goals of their institutions or governments. These teachers are committed to meeting capacity-building goals in resource-poor settings, but their employers insist that they meet institutional goals rather than the goals of the recipient country. The voluntary sector is pretty much excluded from this activity. This "social" model provides an alternative, and this is now aided by the development of new methods of information and communications technology (ICT) that allow not only educational resource production and delivery to occur outside the institutional setting, but also a new way for "students" to collaborate in the learning process with their "teachers" [5]. The "open-source" movement is key to this, where software and educational resource developers give their time to producing and adapting materials which then become freely available on the Internet [6].

The Peoples Open Access Education Initiative – the Peoples-uni – is one of the first examples of this "social" model of international capacity building [7]. Volunteers develop an educational context for resources that are freely available on the Internet, and then deliver this, again using open-source ICT. We report our early experience in attracting volunteers to work on this initiative, and the ability to attract students to the courses. The purpose of this paper is to report on the methods used to develop and deliver this type of capacity-building programme, and the ability to attract volunteers and students.

## Methods

The creation of the Peoples-uni was not based on a scientific approach to the development of a social model of education, as this was a new approach with no previously identified or published methodology. It involved the creation of an administrative infrastructure, calls for and identification of volunteers, development of both the ICT infrastructure and course content, and identification of students and course delivery to them. A pilot course module was a key step, and has been reported [8].

## Results

### Administrative infrastructure

A group of colleagues, known to the author and instigator (RFH), came together to help plan the initiative. After the creation of a charitable trust in the United Kingdom, some of these colleagues became trustees, and others became members of an international advisory group or a quality assurance and educational oversight group, each of which was expanded by further members. All these are volunteers, and other volunteers have subsequently joined to assist with the administrative aspects of running an organization that wishes to provide a high-quality and consistent educational programme. Credibility was gained through the support of the United Kingdom Royal Society for Public Health, which has become an institutional partner and supporter. A small amount of funding has been provided by the United Kingdom Department of Health.

### Course development

#### Choice of courses

Based on many years' experience with the International Clinical Epidemiology Network (INCLEN) [9] and the experience of having developed a fully online Master's-degree course in population health at the University of Manchester [10], we did understand a continuing need for capacity building at the "train the trainers" level. A decision to work initially at this level came through informal discussions with various people in resource-poor settings and a number of specially commissioned situation analyses in countries including the Democratic Republic of the Congo, Ethiopia, India, Nigeria, Sri Lanka and Sudan.

A decision was made to use a competence-based educational model, and to develop modules that covered the "foundation sciences" of public health as well as those that tackled a variety of public health problems. The choice of individual modules was a mixture of availability of potential developers and feedback from students from eight countries enrolled in a pilot-course module on maternal mortality.

The choice of competences has been described previously [8], but was based on discussion among the module developers with a specially developed template and a framework derived from a search for other competences identified in other educational activities aimed at public health practitioners. Open-source materials were readily available to illustrate the chosen competences, although most of these were not originally designed by academics or for formal educational purposes. Access to a number of resources was limited by copyright restrictions, particularly from journal publishers.

A Certificate in Public Health can be obtained on successful completion of any four course modules, and a Diploma in Public Health on successful completion of eight course modules – with at least two from each of the Foundation Sciences of Public Health and the Public Health Problems groups. There are currently 10 course modules in active delivery or development, allowing a choice of modules for the students. Over time, we plan to offer a wider choice as more course modules are delivered.

#### Selection of developers

After the publication of various papers and presentations at meetings as well as personal appeals to colleagues and networks, more than 100 people volunteered to help with course development. They have come from across the globe, in resource-rich and -poor countries.

A development template was devised, based on the course module we had pilot-tested, and this was placed on a special web site using the Moodle open-source platform. The choice of the same development and delivery platform was designed to familiarize the developers with the platform so that they would be ready to act as online facilitators for course delivery. (An alternative of the use of Wiki Educator was explored in the development of the pilot module.)

A majority of those who volunteered, did not in the end make a contribution to the development process. We have not formally investigated the reasons for this, but lack of familiarity with the ICT system, which was perceived to be complex, and lack of clarity in the instructions for its use may have contributed.

To date, 46 people have actively contributed to course module development. Public health trainees from the United Kingdom Faculty of Public Health have provided major input to the development and delivery process, although other active members of the groups are based in 12 other countries. Among these 46 people, 26 were from the United Kingdom and 22 from other countries. As we will discuss below, development is a continuing process, and those involved in the delivery, as tutors and students, are also invited to suggest modifications and additions. A group of ICT students at a United Kingdom university has been commissioned to help us develop a clearer explanation for, and maybe methods of, co-authoring of course modules.

### ICT development

The use of e-learning through the Internet is the basis of the ability of Peoples-uni to assemble an international faculty and deliver courses to people in multiple countries. It also capitalizes on the developments in ICT described above. Some of those involved in the Charity for African Welfare Development and Doctors Worldwide were original supporters of the ICT for Peoples-uni, and they have been joined by others. A server provided by Dasphir, based in Nigeria, hosts the course, and the group has developed a new web site that includes an application system for student applications (with automated enrolments) and student tracking, each linked to Moodle. A system to create, and then for students to gain access to, academic transcripts has also been developed.

### Course delivery

One course module on maternal mortality was pilot-tested at the end of 2007, and six course modules were offered between October 2008 and February 2009. Each module includes five topics designed to last two weeks each, with additional time for catch-up and assignments. Some 29 tutors agreed to act as online facilitators, and 25 have been active. Each course module has one general facilitator to oversee the process, and each topic has one facilitator, who thus may have a role with very limited time commitment. Facilitators are given guides and reminders by the coordinator, but those who have agreed to play an active and identified role have done so. A number of other people have offered to act as online facilitators, and are being asked to join in a future "semester".

### Student numbers

The pilot experience and its feedback are described and are available at . Despite very little publicity or advertising for this initial intake, 117 students enrolled in 170 course modules in October 2008. To keep the discussions manageable, we set a limit of 30 students per module, which resulted in enrolments having to close for all modules (except one) before the end of the enrolment period. Students have come from 23 countries, mainly in Africa, with the largest numbers from Nigeria, Tanzania and Uganda. Seventy-four were male and 43 female; their previous education and current occupation are shown in Table 1.

**Table 1:** Previous education and current occupation of students enrolled in first six course modules

Education and occupation	N (117 in total)
Educational experience	

Medical degree	46

Other health degree	34

Other non-health degree	30

No degree	7

Postgraduate degree (in addition to any above)	25

Current occupation*	

Public health worker	48

Mainly clinician	56

Student	4

Academic	7

*Not known for two students

A fee of USD 50 will be charged for the academic transcript, although a similar amount will be charged in future before the start of the course (by means of an automated payment system). The pilot test revealed that a number of students wanted the knowledge and skills rather than a qualification, and it remains to be seen how many will complete the assignments as required to receive the academic transcripts. As the first set of modules is not fully competed at the time of writing, full information on the follow-up of these students is not available.

## Discussion

The experience with a social model of capacity building in public health for those in resource-poor settings has shown that open-source materials and educational technology to deliver them are readily available, and that volunteers can be mobilized for course development, ICT support, course delivery and administrative infrastructure. An international faculty has been assembled, which includes those in low-resource settings themselves. Health professionals seem keen to enrol as students, but we wait to evaluate the course outcomes. We are in the early stages of the life of the Peoples-uni, and our current capacity to deliver and administer courses is already able to cope with a large uptake of student enrolments.

The development of the Peoples-uni has faced many challenges; some have been overcome and many remain! Among the key challenges are the following.

• First is the identification of volunteers to populate the various functions described in this paper. This has proved successful so far, as we have a sizeable and diverse group in terms of geography and expertise. The sustainability of the initiative will depend on our ability to maintain this volunteer workforce.

• Second is the ability to achieve and maintain quality – for the resources used, the chosen educational model, and the delivery and assessment processes. This has required a series of solutions, including the use of resources from accredited and peer-reviewed sources and the establishment of a group to oversee quality.

• Third is accreditation of the academic awards, which has so far eluded us. We have benchmarked the modules against the European Credit Transfer System, and are in discussions with various organizations about accreditation, but at present the awards are made solely by the Peoples Open Access Education Initiative. Credibility of the awards may depend on our ability to achieve accreditation by other organizations.

• Fourth is the business model, which relies on volunteer activity and a very low enrolment fee. It remains to be seen if this is sustainable.

• The fifth challenge is sustainability, which depends on our ability to meet each of the previous challenges.

This approach is applicable to any academic field, other than those requiring practical skills, such as some aspects of clinical practice. The existence of open educational resources in a wide variety of academic disciplines would allow similar programmes to be developed. For example, the OCW Consortium [11], Rice University Connexions programme [12], and the Open University's OpenLearn initiative [13] have wide ranges of activities for many disciplines. There are other attempts to develop an educational context to Open Educational Resources (OER) in other disciplines – such as the peer-to-peer university [14]. There is a large community of people working on OER [6] and a handbook for educators [15], both of which are relevant for numerous disciplines.

As we improve and adapt the courses, including through the input of students, we are working towards the ability to scale up to accommodate large numbers of students. This will depend on our ability to maintain a large volunteer workforce. While the business model is not fully developed, and the need for some kind of funded infrastructure not clearly identified or articulated, we do believe that the use of the social model of capacity building may allow courses to be offered at a low enough cost to benefit a large number of health professionals who would otherwise not be able to have access to this type of education, and contribute to the public health needs of resource-poor settings. The social model is the result of societal trends towards volunteerism, the availability of the Internet and open-source educational resources and delivery mechanisms. This may not be the only way, but it is our solution to develop a sustainable method of capacity building at low enough cost to meet the requirements of the many people in low-resource settings.

We are also keen to collaborate with educational and other institutions in resource-poor settings, and hope that through their use of Peoples-uni courses and/or joint accreditation of the academic awards, or other forms of collaboration yet to be developed, our educational innovations and international faculty can contribute to the development of these institutions. We encourage anyone who wishes to collaborate in any way to make contact either with the author or through the web site .

## Conclusion

A social model of capacity building in public health has been started and has been able to attract volunteers and students from a wide range of countries. The costs are likely to be low enough to allow this method to make a substantial contribution to capacity building in low-income settings. A number of challenges remain, including the ability to maintain a large volunteer workforce and to build partnerships and collaborations with other organizations.

## Competing interests

The author declares that he has no competing interests.
